# Treatment of Dysmenorrhea with the Galvanic Current

**Published:** 1897-06

**Authors:** 


### TREATMENT OF DYSMENORRHEA WITH THE GALVANIC CURRENT.

Dr. O. A. Gordon (*Brooklyn Medical Journal*, January, 1897) has employed this method of treatment for the past three years, and he believes that for cases not due to a faulty condition of the nervous system, or an impoverished state of the blood, galvanism is the best mode of treatment, and that it will give relief in more cases than any other agent.

Treatment was applied once a week for a month, and then a few days before menstruation for a few times, as follows: A Law battery of sixty cells is employed, and the electrode used resembles an ordinary uterine sound. For the abdomen Dr. G. uses a copper electrode, about four by six inches, and covers it with fresh absorbent cotton for each patient. The uterine electrode is connected with the negative cord, and inserted into the cervical canal, as far as it will go without pressure, and the current turned on until fifteen or twenty milliamperes are reached. Care should be taken that the point of the sound does not rest against the fundus. Five or six minutes is long enough for each application. Antiseptic precautions are observed throughout.

The following advantages, according to the author, may be claimed for this mode of treatment:

1. Patients will submit to electrical treatment when an operation would be declined.

2. It can be carried out in the physician's office without an anesthetic, as it is attended by very little, if any, pain.

3. Any physician with the necessary appliance and moderate knowledge of gynecological work can apply the treatment without assistance.

